# Pancreatic and cardiometabolic complications of severe hypertriglyceridaemia

**DOI:** 10.1097/MOL.0000000000000939

**Published:** 2024-06-06

**Authors:** Bilal Bashir, Maryam Ferdousi, Paul Durrington, Handrean Soran

**Affiliations:** a Faculty of Biology, Medicine and Health, University of Manchester; b Department of Endocrinology, Diabetes and Metabolism, Peter Mount Building, Manchester University NHS Foundation Trust; c NIHR/WELLCOME Trust Clinical Research Facility, Manchester, UK

**Keywords:** apolipoprotein C3, cardiovascular, chylomicronaemia, hypertriglyceridaemia, pancreatitis

## Abstract

**Purpose of review:**

This review endeavours to explore the aetiopathogenesis and impact of severe hypertriglyceridemia (SHTG) and chylomicronaemia on cardiovascular, and pancreatic complications and summarizes the novel pharmacological options for management.

**Recent findings:**

SHTG, although rare, presents significant diagnostic and therapeutic challenges. Familial chylomicronaemia syndrome (FCS), is the rare monogenic form of SHTG, associated with increased acute pancreatitis (AP) risk, whereas relatively common multifactorial chylomicronaemia syndrome (MCS) leans more towards cardiovascular complications. Despite the introduction and validation of the FCS Score, FCS continues to be underdiagnosed and diagnosis is often delayed. Longitudinal data on disease progression remains scant. SHTG-induced AP remains a life-threatening concern, with conservative treatment as the cornerstone while blood purification techniques offer limited additional benefit. Conventional lipid-lowering medications exhibit minimal efficacy, underscoring the growing interest in novel therapeutic avenues, that is, antisense oligonucleotides (ASO) and short interfering RNA (siRNA) targeting apolipoprotein C3 (ApoC3) and angiopoietin-like protein 3 and/or 8 (ANGPTL3/8).

**Summary:**

Despite advancements in understanding the genetic basis and pathogenesis of SHTG, diagnostic and therapeutic challenges persist. The rarity of FCS and the heterogenous phenotype of MCS underscore the need for the development of predictive models for complications and tailored personalized treatment strategies. The establishment of national and international registries is advocated to augment disease comprehension and identify high-risk individuals.

## INTRODUCTION

Triglycerides (TG) represent the predominant components of dietary fat and serve as the primary reservoir of energy. Dietary intake constitutes the primary exogenous origin of TG, with the liver serving as the principal endogenous source. Following ingestion, fats undergo catabolic processes, initiating the clearance of triglyceride-rich lipoproteins (TRL). Nevertheless, under certain circumstances, TG clearance is delayed, attributed to suboptimal lipoprotein lipase (LPL) activity, excessive hepatic production, or compromised hepatic clearance of TRL, resulting in pathological hypertriglyceridemia (HTG). 

**Box 1:**
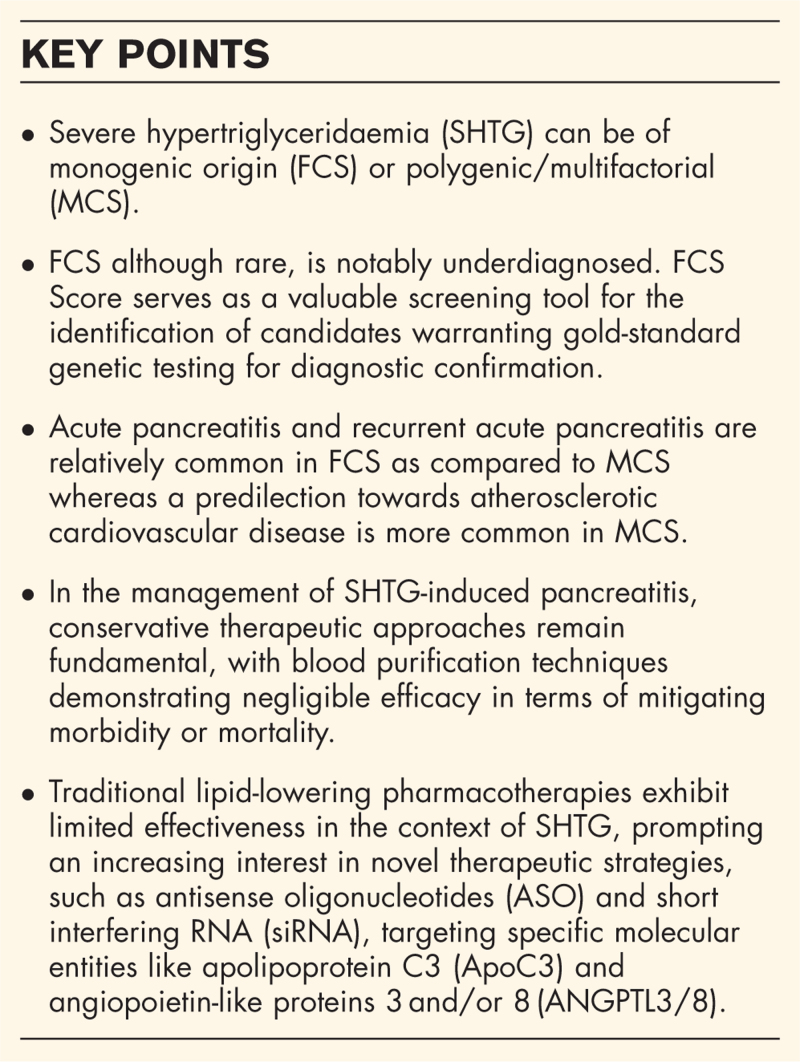
no caption available

### Severity of hypertriglyceridaemia and laboratory measurement

The consensus regarding normal TG concentration is established at < 1.7 mmol/l (150 mg/dl); however, thresholds for classifying the severity of hypertriglyceridemia vary (Table 1) [1–5]. Nonfasting samples offer convenience and comparability with fasting samples for total cholesterol (TC), low-density lipoprotein cholesterol (LDL-C), and high-density lipoprotein cholesterol (HDL-C), whereas TG concentrations tend to be elevated in the postprandial state [6], more so in patients with dyslipidaemia [7], diabetes or insulin resistance [8]. TG correlation with atherosclerotic cardiovascular disease (ASCVD) risk is equally robust for both nonfasting and fasting lipid levels [9]. This association remains robust even after adjusting for other risk factors [10–12]. In the postprandial state, the increment in TRL in systemic circulation is significant, particularly in individuals with diabetes, dyslipidaemia and abdominal obesity that is linked with postprandial heightened endothelial dysfunction, oxidative stress, inflammation and hence atherosclerosis [13]. Therefore, nonfasting lipid profiles are not only useful but potentially more informative than fasting profiles for predicting ASCVD risk. Despite widespread acceptance of nonfasting lipid profile [14–16], one-third of the laboratories across Europe still practice processing fasting samples only for lipid profile [17].

**Table 1:** Definitions of hypertriglyceridaemia

**AHA/ACC**	
Normal	<2.0 mmol/l (174 mg/dl)
Moderate	2.0–5.6 mmol/l (175–499 mg/dl)
Severe	>5.6 mmol/l (>500 mg/dl)
**NCEP ATP III**	
Normal	<1.7 mmol/l (<150 mg/dl)
Borderline high	1.7–2.3 mmol/l (150–199 mg/dl)
High	2.3–5.6 mmol/l (200–499 mg/dl)
Very High	>5.6 mmol/l
**Endocrine Society**	
Normal	<1.7 mmol/l (<150 mg/dl)
Mild	1.7–2.3 mmol/l (150–199 mg/dl)
Moderate	2.3–11.2 mmol/l (200–999 mg/dl)
Severe	11.2–22.4 mmol/l (1000–1999 mg/dl)
Very severe	>22.4 mmol/l (≥2000 mg/dl)
**ESC/EAS**	
Normal	<1.7 mmol/l (<150 mg/dl)
Mild to moderate	>1.7 mmol/l (>150 mg/dl)
Severe	>10.0 mmol/l (>885 mg/dl)

ACC, American College of Cardiology; AHA, American Heart Association; EAS, European Atherosclerosis Society; ESC, European Society of Cardiology; NCEP ATP III, National Cholesterol Education Programme Adult Treatment Plan III.

For ASCVD risk management, LDL-C serves as a primary therapeutic target, commonly calculated using the Friedewald equation. It is suggested to obtain fasting samples for LDL-C quantification when TG concentrations exceed 4.5 mmol/l. However, alternative calculators are available, offering LDL-C estimations up to TG levels of 10 mmol/l [18]. In situations where direct measurement methods (e.g., beta quantification, ultracentrifugation) are unavailable and nonfasting TG levels are exceptionally high, impeding the accurate calculation of LDL-cholesterol, a prudent approach involves obtaining a repeat fasting lipid profile or measuring apolipoprotein B100 (ApoB100) [14,19].

In this review we aim to explore the impact of SHTG, i.e. TG > 10 mmol/l on cardiovascular and pancreatic complications.

### Epidemiology

The prevalence of hypertriglyceridaemia has been reported between 25- 30%. However, the prevalence of severe hypertriglyceridaemia varies depending upon the cut-off used to define it. It is rare and prevalence reduces substantially with rising TG levels. Severe hypertriglyceridaemia, which would be defined for the sake of this review, in line with the definition adopted in the most recent literature as TG > 10 mmol/l, is rare with a projected prevalence of 0.1–0.2% [20^▪^]. Similarly, the prevalence of very severe hypertriglyceridemia, that is, TG concentration exceeding 20 mmol/l is an exceptionally rare phenomenon, with the prevalence of 0.01–0.02%. These reported figures are in line with recent observations made from the largest primary care database in Spain where the prevalence of TG 500–879 mg/dl was 0.7% and ≥880 mg/dl was 0.2% [21].

## AETIOLOGY AND PATHOGENESIS

### Chylomicronaemia syndromes

The term chylomicronaemia syndrome (CS) delineates a distinct subset of SHTG individuals experiencing either intermittent or sustained fasting chylomicronaemia. Whilst abnormalities in catabolism of other TRL subfractions also contribute to severe hypertriglyceridemia, accumulation of chylomicrons represents its distinctive characteristic that is often accompanied by a constellation of symptoms [20^▪^]. Quantifying chylomicron concentration and individual lipoprotein subfractions biochemically is challenging. Therefore, diagnosis of chylomicronaemia often relies on fasting TG levels, which are often >10 mmol/l and observable features, like the milky appearance of plasma, eruptive xanthoma and lipaemia retinalis [22^▪▪^,^23^].

CS can be monogenic, termed familial chylomicronaemia syndrome (FCS), characterized by biallelic loss of function variants in one of the five canonical genes (*LPL*, *APOC2*, *APOA5*, *GPIHBP1* and *LMF1*). Nevertheless, the majority of CS are polygenic, arising from single nucleotide polymorphism (SNP) of multiple common or rare gene variants [22^▪▪^,^24^]. This results in a cumulative impact that increases the susceptibility of individuals to develop chylomicronaemia, termed multifactorial chylomicronaemia syndrome (MCS) [20^▪^].

The precise prevalence of FCS remains a topic of active discussion, with estimates ranging from 1 : 100 000 to 1 : 1 000 000 [25], whereas MCS tends to be more common, 1 : 600–1 : 250 [26]. We contend that FCS is significantly underdiagnosed, and the true prevalence may well exceed the figures presented thus far. Despite distinctive clinical features [20^▪^], the challenge of distinguishing between FCS and MCS often leads to delayed FCS diagnoses [27]. Rapid biochemical tests, such as postheparin LPL activity or lipoprotein electrophoresis, are not widely available and measuring LPL activity is technically demanding. An expert panel introduced an eight-item “FCS Score” to aid the diagnosis of FCS and to better select patients for next-generation sequencing [28]. The score has recently been validated in a large cohort of ethnically and genetically diverse cohort of FCS patients in the UK [29^▪^]. Fasting LDL-C or ApoB100 are also sensitive markers to distinguish FCS from MCS [30]. A higher chylomicron-TG-to VLDL-TG-ratio (CM-TG/VLDL-TG) and TG-to-ApoB100 ratio (TG/ApoB100) is found in the FCS. A CM-TG/VLDL-TG value of 3.8 and 4.5 confers 100% sensitivity and specificity respectively in diagnosis of FCS [31]. Incorporating these biochemical markers with the FCS Score can aid clinicians in accurately predicting the likelihood of FCS, especially in settings with constrained resources for next-generation sequencing genetic testing.

### Familial dysbetalipoproteinaemia

Familial dysbetalipoproteinaemia (FDBL) is characterized by the abnormal accumulation of remnant lipoproteins. Impaired liver processing of chylomicron remnants leads to prolonged circulation of remnant particles and abnormal cholesterol enrichment due to cholesteryl ester transfer protein (CETP) mediated lipid exchanges. Elevated TG and cholesterol levels with a disproportionately low ApoB100 concentration leading to discordances between directly measured and calculated LDL-C are characteristic of FDBL [32]. The condition, often associated with dysfunctional apoE, particularly the E2/E2 genotype, historically has been reported to be causative in 90% of cases of DBL [33]. In a small subset, it is attributed to an autosomal dominant pathogenic variant in ApoE. However recently, Paquette and colleagues challenged this commonly held notion when they found that Apo E2/E2 homozygosity was only present in 38% of FDBL diagnosed by gold standard Fredrickson criteria [34^▪▪^]. A significant correlation was identified between the presence of E2/E2 individuals and the severity of the FDBL phenotype. The E2/E2 subgroup exhibited elevated remnant cholesterol concentrations, low ApoB100, and low LDL-C while demonstrating an increased prevalence of FDBL-related xanthomas compared to the non-E2/E2 group. Moreover, an independent threefold increase in the risk of ASCVD and an 11-fold escalation in the risk of peripheral vascular disease was noted in the E2/E2 group compared to the non-E2/E2 group. The authors suggest subcategorizing FDBL into distinct phenotypes, namely multifactorial remnant cholesterol form and genetic ApoE deficiency form, where genetic ApoE deficiency denotes a more severe phenotype and adverse ASCVD profile [34^▪▪^]. Fredrickson's gold standard criteria for FDBL diagnosis, involving triglyceride levels between 1.7 and 11.3 mmol/l (150–1000 mg/dl) and a VLDL-C/TG ratio >0.69 (0.30 mg/dl) is technically demanding and is not available readily in a clinical setting. However, in the recent past, various simplified criteria and diagnostic algorithms for FDBL have been proposed [35]. Marked elevated ASCVD risk linked to remnant particles and their prolonged circulatory time mandates accurate and early diagnosis along with effective risk stratification in FDBL.

### Lipodystrophies

Lipodystrophies constitute a rare and heterogeneous group of disorders characterized by generalized or partial adipose tissue deficiency. This deficiency leads to metabolic disruptions, including insulin resistance, diabetes, dyslipidaemia, and nonalcoholic steatohepatitis, with a distinctive lipid profile marked by varying degrees of hypertriglyceridemia [36]. The compromised expansibility of adipose tissue exacerbates postprandial lipaemia and triggers lipotoxicity, oxidative stress, mitochondrial dysfunction and endothelial dysfunction.

Congenital generalized lipodystrophy, presenting as an autosomal recessive condition with near-total systemic absence of adipose tissue, results in severe hypertriglyceridemia due to delayed TRL catabolism, primarily linked to profound insulin resistance and reduced LPL activity [37]. Recurrent pancreatitis is common in individuals with suboptimal glycaemic control [38]. Familial partial lipodystrophies (FPLD), rare autosomal dominant disorders, exhibit selective loss of adipose tissue from extremities. The two distinct phenotypes, Dunnigan (type 2) and Köbberling (type 1) variants exhibit an unfavourable metabolic profile including elevated TG, insulin resistance, impaired glucose tolerance, and ASCVD complications [39–41]. The concomitant presence of diabetes results in a heightened prevalence of SHTG and a predisposition for acute pancreatitis [42]. Volanesorsen, licensed for FCS, has shown promise in reducing hepatic steatosis and hypertriglyceridemia in FPLD patients [43^▪^].

### Secondary hypertriglyceridaemia

**FIGURE 1:**
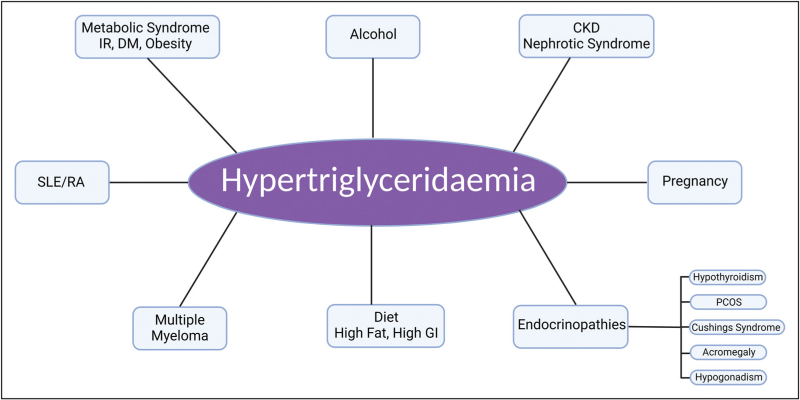
Certain individuals are more predisposed to develop severe hypertriglyceridaemia in the presence of secondary factors. CKD, chronic kidney disease; DM, diabetes mellitus; GI, glycaemic index; IR, insulin resistance; PCOS, polycystic ovarian syndrome; RA, rheumatoid arthritis; SLE, systemic lupus erythematosus.

Several physiological or pathological conditions and medications are frequently, but not universally, associated with varying degrees of hypertriglyceridaemia. Certain specific SNP may render individuals more predisposed to develop SHTG in the presence of these inciting factors. Figure 1 and Table 2 summarize the major causes of acquired hypertriglyceridaemia [20^▪^].

**Table 2:** Drugs with potential to cause hypertriglyceridaemia

Class of drugs	Mechanism
Betablockers^a^	Reduce LPL activity
Thiazide diuretics	Reduce LPL activity
Oral oestrogen	Increase VLDL production
SERM (Tamoxifen)	Increase VLDL production
Corticosteroids	Increase VLDL production
Protease inhibitors	Increase VLDL production and reduce LPL activity
Cyclosporin	Increased ApoC3 levels and reduce LPL activity
Tacrolimus	Reduced LPL activity
1^st^ and 2^nd^ generation antipsychotics	Increase VLDL production, reduce LPL activity
Retinoids	Increased ApoC3 levels
Sirolimus and everolimus	Increased ApoC3 levels
Propofol	Increased fat delivery

Apo, apolipoprotein; LPL, lipoprotein lipase; SERM, selective oestrogen receptor modulator; VLDL, very low density lipoprotein.

a Varies based on individual drug.

## COMPLICATIONS

### Acute Pancreatitis

SHTG is the third leading cause of acute pancreatitis (AP) after gallstones and alcohol. The risk of AP increases progressively with increasing levels of hypertriglyceridaemia[44]. The degree of hypertriglyceridaemia has also been proposed to link with the severity of AP [45–47]. However, it remains uncertain that once hypertriglyceridaemia-induced AP is triggered, the continued existence of chylomicrons extends, sustains or influences the disease course [48^▪^]. In addition to the degree of hypertriglyceridaemia and chylomicronaemia, there are other factors clinical and biochemical variables that govern the severity of acute pancreatitis [49–51].

The exocrine pancreas is a rich source of lipases that liberate a significant quantity of free fatty acids (FFA) by hydrolysis of TG. Once these FFA surpass the binding capacity of albumin, adverse consequences ensue, including mitochondrial toxicity, oxidative stress, endothelial dysregulation, the establishment of a proinflammatory environment, and consequential damage to acinar cells. These detrimental processes ultimately lead to pancreatic acinar necrosis. Moreover, chylomicronaemia and the presence of FFA, facilitated through micelle formation, contribute to an increase in blood viscosity. This hampers blood flow to the pancreas, inducing ischemia. A proportion of pancreatic lipase, instead of entering the gut lumen, navigates into the capillary circulation of the pancreas. Typically inconsequential, this process takes on significance in conditions of chylomicronaemia with sluggish pancreatic microcirculation, allowing the release of cytotoxic fatty acids and lysolipids [20^▪^,^52^].

**FIGURE 2:**
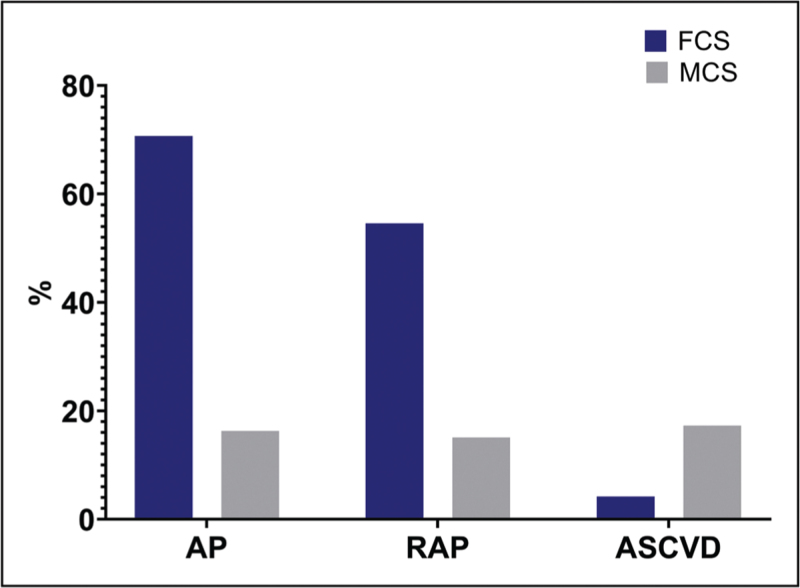
FCS patients are more prone to develop pancreatic and MCS are more prone to develop cardiovascular complications. AP, acute pancreatitis; ASCVD, atherosclerotic cardiovascular disease; FCS, familial chylomicronaemia syndrome; MCS, multifactorial chylomicronaemia syndrome; RAP, recurrent acute pancreatitis.

The lifetime risk of developing AP and recurrent acute pancreatitis (RAP) is higher in FCS as compared to MCS [30,53^▪^,^54^,55^▪^]. The greater propensity to develop acute pancreatitis in FCS could be attributed to sustained, refractory and more severe hypertriglyceridaemia and chylomicronaemia starting at a younger age, more often from infancy. We pooled the results of pancreatic complications from earlier studies and found the prevalence of AP at 70.7% (*n* = 290/410) vs. 16.3% (*n* = 418/2562) and RAP at 54.6% (*n* = 202/370) vs. 15.1% (*n* = 49/324) in FCS as compared to MCS [30,31,54,55^▪^,^56^–^58^] (Fig. 2). We have reported a similar trend but a higher proportion of AP and RAP along with earlier age of symptoms and AP onset with a significant genetic heterogeneity from a cohort of patients from the UK FCS National register [59^▪▪^]. In MCS, there has been some recent evidence suggesting an elevated genetic load for pancreatitis susceptibility genes is linked to an increased risk of pancreatitis [60]. In addition to maximum TG concentration, HbA1c, GGT and low HDL, the presence of a heterozygous LOF variant in one of the canonical genes is a strong predictor of AP and RAP in MCS [54]. In a recent study by Deshotels *et al.*, individuals with both heterozygous rare pathogenic variants and high polygenic scores had significantly higher TG and a fivefold increased risk of AP as compared to those without pathogenic variants and low polygenic scores. Interestingly, authors did not find the risk of severe hypertriglyceridaemia (>10 mmol/l) and risk of pancreatitis significantly different in patients with a pathogenic variant and low polygenic score group and no pathogenic variant and high polygenic score as compared to patients with no pathogenic variant and low polygenic score [61].

#### Management

Management guidelines for hypertriglyceridemia-induced pancreatitis (HTGP) remain lacking, with standard treatment primarily involving the cessation of oral intake or the provision of fat-free enteral or parenteral nutrition to halt chylomicron production. In severe cases, clinicians often supplement these measures with interventions such as insulin infusion or blood purification techniques, aiming to rapidly reduce TG. However, these additional interventions, including blood purification techniques, have not demonstrated sustained long-term improvements in HTGP outcomes [20^▪^]. Although blood purification techniques effectively lower TG levels within 24 h, this effect is not sustained beyond 72 h when re-bound hypertriglyceridaemia can ensue. Additionally, they fail to confer discernible benefits regarding systemic inflammation, length of hospital stay, local complications, or mortality compared to conventional or insulin therapy. However, it may increase the risk of complications and hospital costs [62^▪▪^,^63^,64^▪^,^65^,^66^], potentially influenced by a selection bias favouring intervention in severely ill patients. In the only randomized controlled trial (RCT) comparing plasma exchange with insulin infusion in mild HTGP, neither modality demonstrated superiority in rapidly reducing TG levels or improving clinical outcomes [67^▪▪^]. An RCT is essential to conclusively determine the utility of blood purification modalities in HTGP. In a recent preplanned analysis of The Effect of Plasma Triglyceride-Lowering Therapy on the Evolution of Organ Function in Early Hypertriglyceridemia-Induced Acute Pancreatitis Patients With Worrisome Features (PERFORM Study), investigating the impact of blood purification techniques on early HTGP patients with concerning features, it was found that blood purification methods did not provide additional benefits in terms of mitigating organ failure, pancreatic complications, or mortality [68^▪▪^]. In the propensity score matched cohort, half of the study cohort had APACHE II score ≥8 and both arms were well matched on other characteristics. Based on a comprehensive review of current literature, a judicious approach would involve initiating conservative treatment initially. In the absence of strong evidence to support the use of blood purification techniques, their use should be reserved for patients displaying persistent deterioration, but treatment should be individualized [69]. We have recently proposed a management algorithm for HTGP where the cornerstone in the management of HTGP stayed conservative treatment with invasive intervention reserved only for patients who continue to show signs of deterioration [20^▪^].

### Atherosclerotic cardiovascular disease

TRL breakdown yields remnant particles, small dense LDL particles, HDL3, and FFA. FFAs induce endothelial dysfunction via oxidative stress, reduced nitric oxide (NO) production, inflammation, and endothelial cell apoptosis [70]. Remnant particles, smaller with higher cholesterol-to-triglyceride ratios, persist longer in circulation owing to reduced LPL or reduced hepatic uptake. Prolonged circulation along with their preferential entrapment and residence in vascular endothelium coupled with uptake by macrophages without the need for chemical modification accelerates atherosclerosis [71]. Apolipoprotein B48 (ApoB48), present in chylomicron remnants, is found in atherosclerotic plaques, implicating it in plaque formation. Historically, scepticism about chylomicronemia's role in atherosclerosis stemmed from large chylomicron particles presumed unable to penetrate the vascular intima [72,73]. However, SHTG often involves smaller remnant particles, facilitating their entrapment in blood vessels and subsequent macrophage uptake, triggering an inflammatory response conducive to atherogenesis.

Although hypertriglyceridaemia has been demonstrated to be instrumental in conferring residual cardiovascular risk in some recent population-based studies [74^▪^,^75^,^76^], the association of SHTG with ASCVD has been difficult to establish. The rarity of SHTG, the occurrence of its primary form at a young age, and the predominant focus on the higher prevalence of life-threatening AP have hindered a thorough examination of the risk of ASCVD. A recent population-based study from USA, compared to normal TG (<1.7 mmol/l) hypertriglyceridemia (2.2–5.6 mmol/l) at optimum LDL-C (1.0–2.6 mmol/l), was found to increase the risk of nonfatal MI, nonfatal stroke and coronary revascularization [relative risk (RR) 1.30, (1.08–1.58), 1.23 (1.01–1.49) and 1.21(1.02–1.43), respectively] [77]. Similar observations were made by a subsequent study from Italy whereas compared to normal TG (<1.7 mmol/l), TG of 1.7–5.6 mmol/l and >5.6 mmol/l, after adjusting for confounders, were associated with a higher risk of incident ASCVD [hazard ratio (HR) 1.61 (1.43–1.82) and 2.30 (1.02–5.18), respectively] and all-cause mortality [HR 1.49 (1.36–1.63) and HR 3.08 (1.46–6.50), respectively] [76]. A recent population-based study in England revealed an elevated risk of myocardial infarction (MI) among individuals with SHTG in a univariate model [TG 10–20 mmol/l; HR 1.85 (1.60–2.15), TG > 20 mmol/l; HR 2.04 (1.49–2.78)]. However, upon adjusting for other covariates, this heightened risk failed to remain statistically significant in SHTG [TG 10–20 mmol/l; HR 1.03 (0.88–1.19), TG > 20 mmol/l; HR 1.02 (0.75–1.40)]. Consistent with previous findings, individuals with mild and moderate hypertriglyceridemia exhibited an increased risk of acute MI. All-cause mortality showed a gradual increase with the severity of hypertriglyceridemia [74^▪^].

Monogenic form of SHTG, that is, FCS patients exhibit defective LPL-mediated lipolysis, resulting in the accumulation of circulating chylomicrons rather than atherogenic remnants. Moreover, patients with FCS tend to have relatively healthy cardiometabolic risk profiles. Therefore, ASCVD occurrence is not common and tends to prevail later in life in FCS (Fig. 2).

### Microvascular Complications

The independent impact of hypertriglyceridemia on microvascular complications is intricate due to confounding factors such as diabetes and obesity, which are not only independent risk factors for retinopathy, neuropathy, and nephropathy but also contributors to hypertriglyceridemia. Hypertriglyceridemia was associated with a 23% increased risk of retinopathy progression and development of high-risk proliferative diabetic retinopathy (PDR) in The Early Treatment Diabetic Retinopathy Study (ETDRS) [78], although its independent association with incident nonproliferative diabetic retinopathy (NPDR) has not been demonstrated [79,80]. Neuropathy, including distal diabetic symmetrical peripheral neuropathy and cardiac autonomic neuropathy, is associated with hypertriglyceridemia [81]. We have demonstrated significant small nerve fibre damage in patients with TG > 5.5 mmol/l in patients without diabetes [82^▪▪^]. Interestingly, regeneration of corneal small nerve fibre in obese patients with [83] and without [84] type 2 diabetes after bariatric surgery correlated with improvement in TG level but not fasting glucose or HbA1c.

Lipoprotein glomerulopathy (LPG), characterized by lipid thrombi deposition in glomerular capillaries due to severe hypertriglyceridemia, was observed, particularly in FDBL patients but also observed in SHTG of other aetiologies [85–87]. Proteinuria tends to improve with a low-fat diet-induced reduction in TG [85]. High-fat diets promoting hypertriglyceridemia were associated with an increased risk of chronic kidney disease (CKD) [88], with similar pathogenic mechanisms implicated in other microvascular and macrovascular complications.

## MANAGEMENT

### Diet, lifestyle and secondary factor management

Diet and lifestyle management along with optimization of secondary factors (Fig. 1) remain the cornerstone in the management of SHTG and chylomicronaemia. A very-low-fat diet comprising <15% of total caloric intake or <15–20 g of fat is recommended. Reducing the intake of refined carbohydrates, alcohol, and high-fat meals along with emphasis on fat-free dairy alternatives and incorporating nutrient-rich options such as leafy greens, whole grains, flaxseeds, soybeans, tofu, or walnuts to fulfil essential fatty acid needs is recommended. Supplementation with fat-soluble vitamins is also advised [89]. Intensification of lifestyle modification, exercise, elimination of secondary factors and weight loss improves hypertriglyceridaemia via multifaceted approaches [90]. Despite benefits, adherence to low-fat diets presents challenges, leading to psychosocial issues such as depression and eating disorders. Patients often struggle to comply due to social and emotional pressures [27]. Personalized dietary guidance and continuous monitoring are essential components of managing severe hypertriglyceridemia.

### Pharmacotherapy

Conventional lipid-lowering medications used to lower lipid levels often have limited efficacy in cases of very SHTG, in particular patients with FCS. RCTs have primarily focused on patients with mild to moderate hypertriglyceridemia, with ASCVD as the main endpoint. The drug classes targeting serum TG, including fibrates, omega-3 fatty acids, orlistat and niacin, typically achieve reductions of 25–50% in triglyceride levels among those with mild to moderate hypertriglyceridemia. However, these medications are insufficient to reduce TG to a safe level when applied to severe hypertriglyceridemia to prevent AP.

#### Novel Agents

Certain innovative therapeutic approaches have emerged in the recent past, notably including antisense oligonucleotides (ASO) and small interfering RNA (siRNA) targeting APOC3 and ANGPTL3/8 implicated in TG metabolism. These novel pharmacotherapeutic agents hold promise not only in effectively addressing elevated TG concentrations but also in reducing the incidence of AP and are at various stages of development (Table 3) [20^▪^,91^▪^,^92^,^93^^▪▪^,^94^–^97^,^98^^▪▪^,^99^^▪▪^,^100^–^107^,^108^^▪▪^].

**Table 3:** Emerging pharmacotherapy for severe hypertriglyceridaemia

Agent	Mechanism of action	Dose	Effect on TG	Phase of development	Comments
**ApoC3 inhibitors**					
Volanesorsen	ASO against hepatic ApoC3	285 mg weekly for 3 months followed by fortnightly	50–70% reduction	Approved by EMA and NICE for use in FCS in 2019	Thrombocytopenia remains a predominant side effect requiring close monitoring. Not advisable to start if the platelet count is <140 × 10^9^/l
Olezarcen	GalNAc3 conjugated ASO against hepatic ApoC3	80 mg monthly	45–60% reduction	Phase III trial completed	Targets ASGPR in hepatocytes with efficacy comparable to native ASOs at 20–30-fold lower dosage, thereby minimizing side effects such as thrombocytopenia
Plozasiran ARO APO CIII	siRNA against ApoC3	50 mg every 3 months	Upto 90% reduction	Phase III trial (PALISADE) is ongoing and expected to be completed in 2026.	In phase, I, along with TG reduction, a dose-dependent increase in HDL was also observed
**ANGPTL3 inhibitors**					
AROANG3	siRNA against ANGPTL3	Variable 35–300 mg	Upto 50% reduction	Phase II trials (ARCHES-2) is ongoing and expected to be completed in December 2024	Also results in upto 50% reduction in LDL-C. Planning for Phase III of GATEWAY (study in patients with HoFH) is underway
Evinacumab	Monoclonal antibody against ANGPTL3	15 mg/kg 4 weekly	Upto 80%	No further development for sHTG	Phase 2 study found no TG reduction in patients with biallelic LOF variants in FCS-causing genes. Cohorts with single LOF variants or no gene variants showed 64.8% and 81.7% TG reductions, respectively
LY3561774	siRNA against ANGPTL3	Phase 1 trial completed, results not published yet.			
LY3475766	Monoclonal antibody against ANGPTL3/8 complex	100–600 mg single dose	Upto 70% (dose dependant)	Phase I completed	No further updates since April 2021
**Suspended therapies**					
Alipogene Tiparvovec (Glybera)	Gene replacement	-	40–60% reduction initially.	Approved by EMA in 2012 for clinical use but withdrawn from the market owing to poor commercial prospects in 2017.	Sustained gene expression and reduced risk of pancreatitis despite the transient effect on hypertriglyceridemia
Vupanorsen	ASO against ANGPTL3	-	50–60% reduction	Development halted in 2022 after a review of the Phase 2b (TRANSLATE-TIMI) study	Data from the Phase 2b trial did not support the clinical development of the drug for CV risk reduction or sHTG. It was also associated with dose-dependent hepatotoxicity
STT-5058	Monoclonal antibody against ApoC3	Phase I trial was terminated due to recruitment failure			

ANGPTL 3, angiopoietin like 3; AP, acute pancreatitis; ASGPR, asialoglycoprotein receptors; ASO, antisense oligonucleotide; DAG, diacylglycerol transferase; EMA, European Medicine Agency; FCS, familial chylomicronaemia syndrome; GALNAc_3_, *N*-acetyl galactosamine; HoFH, homozygous familial hypercholesterolemia; SHTG, severe hypertriglyceridemia; siRNA, small interfering RNA; TRANSLATE-TIMI, targeting ANGPTL3 with an antisense oligonucleotide in adults with dyslipidaemia.

## CONCLUSION

Due to the rarity of the condition, challenges continue to exist regarding diagnostic ambiguity, underdiagnosis and misdiagnosis due to asymptomatic presentation or symptom misattribution. Additionally, the genetic intricacies associated with FCS and MCS pose distinctive challenges, necessitating advanced techniques for variant identification and comprehension of their metabolic implications. The phenotype of MCS is heterogeneous, where factors governing heterogeneity are largely unknown. Current therapeutic options remain limited, highlighting the necessity for novel treatments targeting specific pathways. To date, the predominant focus of SHTG has been on reducing the risk of AP and its implications on macrovascular and microvascular disease have not been studied. Due to the rarity of the disease, the establishment of national and international registries, particularly for the monogenic form of the disease is essential to untangle the natural history of the disease and to identify patients at the highest risk of complications.

## Acknowledgements

*The authors acknowledge the support from the National Institute for Health Research, WELLCOME Clinical Research Facility, Manchester Biomedical Research Centre, Lipid Disease Fund and Hyperlipidaemia Education & Atherosclerosis Research Trust UK (HEART UK).*

*Author contributions:*

*B.B. performed literature searches, wrote the first and edited the subsequent drafts.*

*M.F., P.D. contributed to literature search writing and revising the drafts.*

*H.S. conceptualized this review, revised the drafts, and completed the final review.*

### Financial support and sponsorship

*None.*

### Conflicts of interest

*There are no conflicts of interest.*

## REFERENCES AND RECOMMENDED READING

Papers of particular interest, published within the annual period of review, have been highlighted as:

▪ of special interest
▪▪ of outstanding interest
